# Causes of acute respiratory hospitalizations predict survival in fibrosing interstitial lung diseases

**DOI:** 10.1371/journal.pone.0242860

**Published:** 2020-11-30

**Authors:** Johanna Salonen, Hannu Vähänikkilä, Minna Purokivi, Riitta Kaarteenaho

**Affiliations:** 1 Respiratory Medicine, Research Unit of Internal Medicine, University of Oulu, Oulu, Finland; 2 Medical Research Center (MRC) Oulu, Oulu University Hospital, Oulu, Finland; 3 Infrastructure for Population Studies, University of Oulu, Oulu, Finland; 4 The Center of Medicine and Clinical Research, Division of Respiratory Medicine, Kuopio University Hospital, Kuopio, Finland; BronxCare Health System, Affiliated with Icahn School of Medicine at Mount Sinai, NY, USA, UNITED STATES

## Abstract

Acute exacerbation of ILD (AE-ILD) is a common reason for hospitalization; it is also associated with significant mortality. Less is known about the prognostic significance of other events causing acute, non-elective hospitalizations in ILD patients. ILD patients hospitalized due to acute respiratory worsening were collected from medical records. Reasons for respiratory deterioration were classified into AE-ILDs and other causes. Clinical features and survival data of idiopathic pulmonary fibrosis (IPF) and other types of ILDs were evaluated and compared. In all, 237 patients (138 with IPF and 99 with other ILD) fulfilled the inclusion criteria. Of the non-IPF ILD types, the most prevalent subgroups were connective tissue disease-associated ILD (n = 33) and asbestosis (n = 22). The most common cause for hospitalization was AE-ILD explaining 41% of hospitalizations. Lower respiratory tract infection (22%), subacute progression of ILD (12%) and cardiovascular causes (7.2%) were other common reasons for hospital treatment. Patients with a lower respiratory tract infection had a more favorable prognosis compared with patients with AE-ILD. AE-ILDs were less fatal than cardiovascular or concurrent non-ILD-related causes for hospitalizations in non-IPF patients. High Gender-Age-Physiology (GAP) index was a marker for shortened survival and earlier AE-ILDs in all patients. IPF patients had a significantly shorter overall and post-hospitalization survival time compared with other ILDs. Most respiratory hospitalizations in ILD patients were related to causes other than AE-ILD, which highlights the importance of accurate differential diagnosis in order to target the appropriate treatment for each ILD patient.

## Introduction

Idiopathic pulmonary fibrosis (IPF) and other progressive fibrosing interstitial lung diseases (ILD) are serious, often fatal conditions also causing a high burden for health care systems. In a Spanish study, the numbers of hospitalizations related to IPF almost doubled between 2004 and 2013 from 3.82/100 000 inhabitants to 6.98/100 000 inhabitants [1].

Based on the results of the epidemiological studies, a significant proportion of hospitalizations in ILD patients were related to ILD or other reasons causing respiratory symptoms [1–4], and the majority of ILD patients had experienced some kind of event requiring hospitalization during their disease history [4]. Most of the register-based studies have investigated solely patients with IPF, while those of other ILDs are sparse. Table 1 presents data from previously published epidemiological studies and other studies on hospitalizations of ILD patients [5–11].

**Table 1:** Previous studies investigating hospitalizations of patients with interstitial lung diseases (ILD).

Study	Setting	Number of patients and/or treatment periods	Causes for hospitalization (%)
Epidemiologic studies based on diagnosis codes without an evaluation of clinical data			
Yu et al., 2016, USA [2]	Commercial administrative claims data between 2006–2011, 1-year follow-up. ICD-9-CM code 516.3 and additional criteria used to identify IPF patients.	1735 patients (516.3)	Proportions of patients:
			All-cause 38.6%
			IPF-related 10.8%
Cottin et al., 2017, France [3]	French national hospital discharge database between 2008–2013. Over 50-year-old patients with ICD-10 code J84.1	6476 patients (J84.1) 16 106 treatment periods	Proportions of patients:
			Acute event (respiratory or other) 87%
			Respiratory infection 43.7%
			Acute respiratory worsening 36.5%
			Cardiac event 51.7%
Pedraza-Serrano et al. 2017, Spain [1]	Hospitalized patients from the Spanish National Hospital Database between 2004–2013. Patients with ICD-9-CM code 516.3	22 214 treatment periods (516.3)	Most common diagnoses combined with ICD-9-CM 516.3 (proportions of treatment periods):
			Acute and chronic respiratory failure 18.4%
			Other disease of respiratory system 14.3%
			Acute respiratory failure 13.2%
			Pneumonia 6.4%
			Heart failure 5.1%
Wälscher et al., 2020, Germany [4]	German claims data from 2009 to 2014. Patients with ICD-10 codes J84.1, J84.0, J84.9, D48.1, D86.0-D86.9, J70.2-J70.4, J62.0-J62.8, J63.2, J70.1, J82, J67.9 and J99.1.	154 109 hospitalizations	Proportions of patients:
		14 453 patients (J84.1)	ILD-related 56.6%
		22 364 patients with other codes	Non-ILD-related 71.2%
Retrospective studies investigating hospitalizations of patients with an evaluation of clinical data			
Moua et al., 2016, USA [5]	Retrospective data on ILD patients hospitalized due to acute respiratory worsening in one center between 2000 and 2014.	311 hospital admissions	Proportions of hospital admissions:
		100 IPF patients	AE-ILD 52%
		120 Non-IPF patients	Infection 20%
			Subacute progression 15%
			Cardiac 6%
			Thromboembolic 4%
			Multifactorial <1%
Teramachi et al. 2018, Japan [10]	Retrospective data on IPF patients with respiratory hospitalization from one center between 2008 and 2017.	122 IPF patients	Data concerning first hospitalization:
			AE-IPF 29%
			Subacute progression 17%
			Pneumonia 23%
			Lower respiratory tract infection 9%
			Other parenchymal cause 9%
			Extra-parenchymal cause 13%
Song et al., 2011, South Korea [6]	Retrospective data on IPF patients from one center between 1990 and 2009	461 IPF patients	Proportions of patients:
			Respiratory (total) 35.4%
			AE-IPF 19.5%
			Lower respiratory tract infection 11.1%
			Heart failure 1.1%
Yamazaki et al., 2020, Japan [11]	Retrospective data on IPF and chronic idiopathic interstitial pneumonia (c-IIP) patients with respiratory hospitalizations from one center between 2008 and 2018.	138 IPF patients	Proportions of patients (first hospitalization):
		105 c-IIP patients	
		Total 243	AE-ILD 48%
			Pulmonary infection 33%
			Pneumothorax and/or mediastinal emphysema 10%
			Heart failure 3.3%
Ratwani et al., 2019, USA [7]	Retrospective data on connective tissue disease associated interstitial lung diseases (CTD-ILD) patients from one center between 2010 and 2017	137 CTD-ILD patients	Proportions of patients:
			No hospitalizations 32%
			Cardiopulmonary 51%
			Non-cardiopulmonary 17%
			AE-ILD: NA
Behr et al., 2015, Germany [8]	Data from multicenter national INSIGHT-IPF-registry collected between 2012–2014	502 IPF patients	Proportions of the patients hospitalized within the last 12 months:
			IPF-related 42.9%
			Non-IPF-related 3.9%
			AE-IPF: NA
Brown et al., 2015, USA [9]	Retrospective data on IPF patients from one center between 1997 and 2012.	592 IPF patients	Proportions of patients:
			No hospitalizations 74.7%
			Respiratory 19.6%
			Non-respiratory 5.7%
			AE-IPF: NA

Abbreviations: AE-ILD, acute exacerbation of interstitial lung disease; AE-IPF, acute exacerbation of idiopathic pulmonary fibrosis; c-IIP, chronic idiopathic interstitial pneumonia; CTD-ILD, connective tissue disease-associated interstitial lung disease; ICD-9CM, International Classification of Diseases, ninth revision, Clinical Modification; ICD-10, International Classification of Diseases, version 10.

Acute exacerbation (AE) is a severe complication of ILD, which seems to cause significant mortality in all types of ILDs [12, 13]. The current diagnostic criteria for AE-IPF have been suggested to be applied for other ILDs as well [12, 13]. The diagnosis of AE-ILD requires a detailed evaluation of clinical symptoms and high-resolution computed tomography (HRCT) images of chest [12]. One substantial problem in AE-ILD research is the fact that no specific diagnosis code exists for AE-ILD. As a consequence, the large epidemiological studies mentioned above do not provide detailed information on the number of AE-ILDs [1–4]. In other studies with access to the patient data, the proportion of AE-ILDs out of all the hospitalizations caused by an acute respiratory deterioration has ranged between 29–55% [5, 6, 10, 11], when only two of these investigations included patients with non-IPF ILDs [5, 11]. In the previous studies, which have not presented the exact data on the number of AE-ILDs, it was claimed that respiratory or cardiopulmonary hospitalizations increased the mortality of ILD patients more than hospitalization due to other illnesses [7, 9].

The aim of this present study was to evaluate fibrosing ILD patients hospitalized non-electively due to an acute deterioration of respiratory symptoms in two Finnish hospitals, namely Oulu University Hospital (OUH) and Oulaskangas Hospital (OH). The causes for hospitalizations were evaluated and classified as either AE-ILDs or other reasons, the risk factors for mortality and AE-ILDs were compared between IPF and non-IPF patients.

## Material and methods

### Patient and data collection

**Fig 1:**
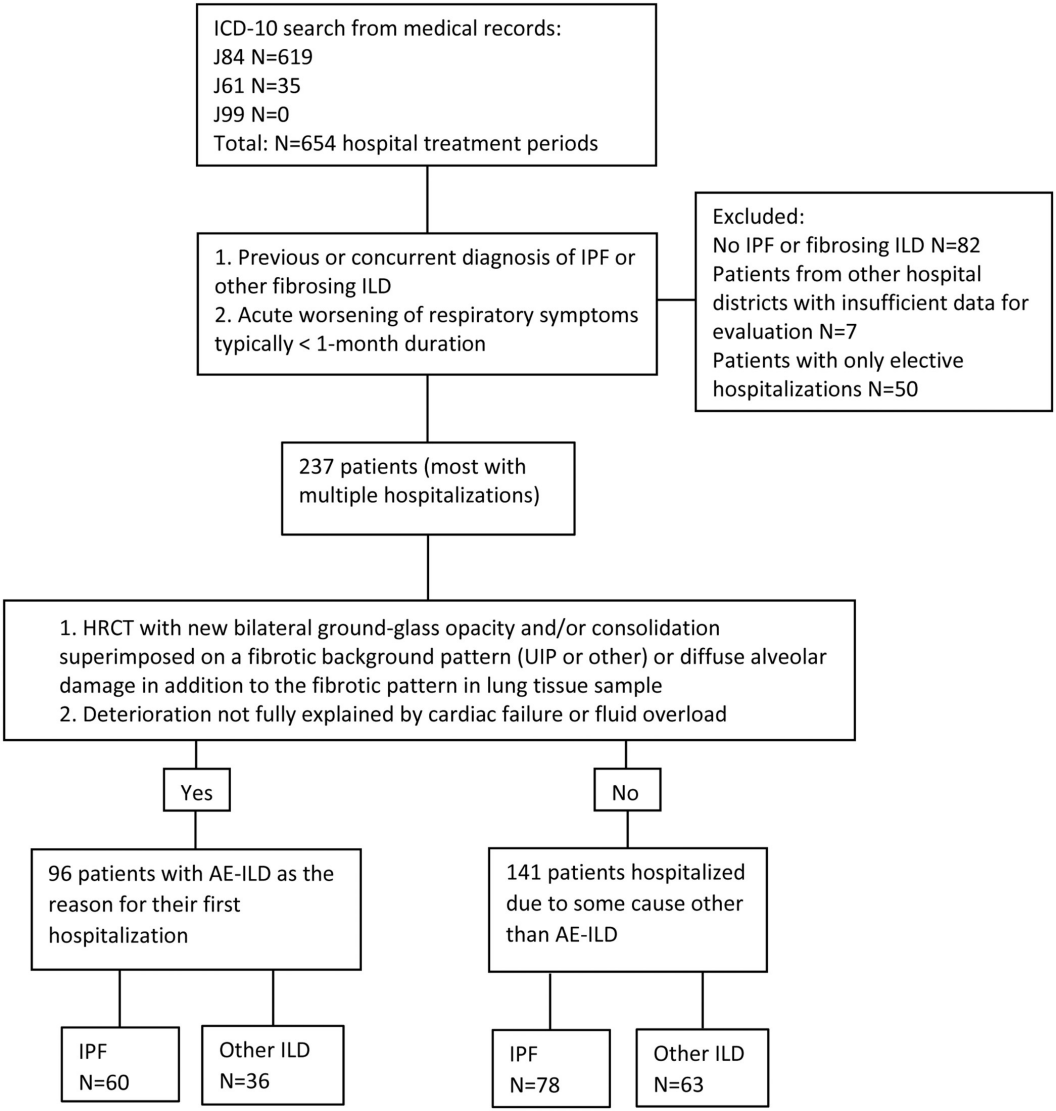
Flowchart of the study.

The study material consists of ILD patients who were non-electively hospitalized due to acute respiratory symptoms in OUH or OH between 1/1/2008 and 31/12/2017. A total of 128 of the 237 patients included were also included in our previous study [14]. The flowchart of the study and criteria used to define AE-ILD are presented in Fig 1. For definition of AE-ILD, we applied the current criteria by Collard et al., thus including both triggered and idiopathic AE-ILDs [12]. We performed a search with International Classification of Diseases version 10 (ICD-10) codes J84.1, J84.8 and J84.9 to identify the patients and their treatment periods from medical records as described previously [14]. Another search was performed with ICD-10 codes J61, J99, J99.0* and J99*M05.1 to find the hospitalizations related to asbestosis and rheumatoid arthritis-associated ILD (RA-ILD). However, there were no matches for the search with J99 codes, because J84 codes had been used for all RA-ILD patients in our cohort. Most ILD patients had undergone a multidisciplinary diagnosis (MDD) evaluation by pulmonologists, thoracic radiologists and a lung pathologist. For this particular study, the type of ILD was re-evaluated according to the current international criteria assessing all clinical data from patient records and death certificates, pathologists’ reports and the radiologists’ reports [15, 16]. The re-evaluation was performed by a specialist physician in pulmonary medicine (JS). Ambiguous cases were re-assessed by another specialist physician in pulmonary medicine (RK) participating in this study. All patients included (n = 237) had HRCT images of chest available for evaluation of ILD type. The clinical information was collected systematically from electronic patient records dating back about 20 years in OUH and OH in a form designed for the present study. We collected the date of birth, age at diagnosis and at hospitalization, gender, smoking status, date of ILD diagnosis, pulmonary function test (PFT), HRCT reports and images, use of long-term oxygen treatment, pharmacological therapy prior to hospital admission and the possible trigger for AE-ILD. Patients with less than a 5 years’ smoking history were considered non-smokers. Recorded PFT values were measured maximally 6 months prior to or after the diagnosis or hospitalization date. PFT was performed according to the ATS/ERS guidelines [17, 18] and Finnish reference values were used for interpretation of the results [19]. The following equipment was used in evaluating PFTs; Jaeger Masterscreen PFT (2008–2013), Carefusion Masterscreen PFT SeSu (2013–2016) and from 2016 on Carefusion Masterscreen RT SeSu and Jaeger MasterScreen™ PFT specifications (with JLab) were used. The PFT devices have been regularly updated according to the ATS/ERS guidelines and reference values by the equipment manufacturers.

We categorized the reasons for hospitalizations to AE-ILDs and other reasons having caused respiratory symptoms, as presented in Fig 1. The detailed data on hospitalizations was collected related to the first non-elective hospitalization after or at the time of ILD diagnosis, if the diagnosis of ILD was made during a non-elective hospitalization. Out of the 237 patients included in the study, there were 96 patients whose first respiratory hospitalization was caused by an AE-ILD (Table 2). We also evaluated the subsequent respiratory hospitalizations of those patients who had their first respiratory hospitalization due to a cause other than an AE-ILD to identify the patients who experienced an episode of AE-ILD later. Almost half, i.e. 109 patients out of 237 did not experience an AE-ILD at all during the follow-up time. Thirty-two patients out of 237 who were first hospitalized due to some reason other than AE-ILD, were discharged and experienced a hospital treatment caused by an AE-ILD later during the follow-up time. The date of the first AE-ILD of each patient having experienced this episode was recorded and utilized for the evaluation of the risk for AE-ILD.

**Table 2:** Characteristics of the patients hospitalized due to acute respiratory symptoms.

Characteristic	Total	IPF	Other ILD	P-value
	N = 237	N = 138	N = 99	
Age at diagnosis (years)	69±11	70±10	68±13	0.155
Age at hospitalization (years)	73±9.7	73±9.7	74±9.8	0.603
Gender male	144 (61)	91 (66)	53 (54)	0.054
Histopathology of ILD				
No histopathology available	167 (71)	100 (73)	67 (68)	0.426
Surgical lung biopsy	34 (14)	19 (14)	15 (15)	0.764
Autopsy	28 (12)	15 (11)	13 (13)	0.595
Surgical lung biopsy and autopsy	8 (3.4)	4 (2.9)	4 (0.4)	0.722
PFT at diagnosis				
VC (% of pred) ^a^	72 ±16	70±15	76±17	0.012
FVC (% of pred) ^b^	74±16	72±15	76±17	0.090
FEV1 (% of pred) ^b^	78±17	77±16	79±19	0.225
FEV1/FVC ^c^	85±9.0	86±10	85±7.0	0.419
FEV1/FVC (% of pred) ^c^	106±9.3	107±8.9	105±9.4	0.028
DLCO (% of pred) ^d^	53±18	48±17	58±18	<0.001
PFT at hospitalization				
VC (% of pred) ^e^	60±17	59±15	62±19	0.340
FVC (% of pred) ^f^	63±18	62±16	65±20	0.297
FEV1 (% of pred) ^g^	68±17	68±15	69±20	0.650
FEV1/FVC ^h^	87±6.7	88 ±6.3	85±6.9	0.012
FEV1/FVC (% of pred) ^h^	108±9.2	109±8.9	107±9.5	0.061
DLCO (% of pred)^i^	41±14	39±14	43±15	0.106
GAP at diagnosis^j^	3 (2–5)	4 (3–5)	3 (2–4)	0.002
Stage I (0–3 points)	109 (51)	51 (43)	58 (62)	0.004
Stage II (4–5 points)	79 (37)	52 (43)	27 (29)	0.032
Stage III (6–8 points)	25 (12)	17 (14)	8 (8.6)	0.211
No former ILD diagnosis	62 (26)	37 (27)	25 (25)	0.788
AE-ILD during follow-up	128 (54.0)	79 (57.2)	49 (49.5)	0.238
First hospitalization due to AE-ILD	96 (40.5)	60 (43.5)	36 (36.4)	0.271
First hospitalization due to reason other than AE-ILD	32 (13.5)	19 (13.8)	13 (13.1)	0.887
Smoking at hospitalization ^k^				
Current smoker	16 (6.9)	11 (8.2)	5 (5.1)	0.356
Ex-smoker	100 (43)	62 (46)	38 (39)	0.255
Non-smoker	116 (50)	61 (46)	55 (56)	0.111
Pack-years of ever-smokers ^l^	27±17	29±18	24±14	0.169
Long-term oxygen therapy at home before hospitalization ^m^	29 (12)	17 (13)	12 (12)	0.931
Time from diagnosis to hospitalization (excluding first time diagnosis), years	3.4 (1.2–8.1)	2.4 (0.9–5.5)	5.9 (2.3–10.7)	<0.001
Follow-up time, years	4.7 (1.9–9.0)	3.5 (1.4–6.6)	6.6 (3.6–11.1)	<0.001
Time from hospitalization to last follow-up date, months	16.5 (2.6–42.9)	13.5 (1.9–29.8)	22.0 (4.4–58.9)	0.008
1st hospitalization led to death	26 (11)	16 (12)	10 (10)	0.717
Deceased during the follow- up	206 (87)	127 (92)	79 (80)	0.006
Lung transplantation	7 (3.0)	5 (3.6)	2 (2.0)	0.702

Data is expressed as numbers of patients (%), means (±standard deviation) or medians (interquartile range). Changes in PFT results were calculated as follows: (PFT at hospitalization (absolute value) minus PFT at diagnosis (absolute value)) divided by PFT result (absolute value) at diagnosis. Those patients who were diagnosed with ILD during the hospital treatment period were excluded.

^a^ Data of 50 patients was missing.

^b^ Data of 19 patients was missing.

^c^ Data of 22 patients was missing.

^d^ Data of 29 patients was missing.

^e^ Data of 63 patients was missing.

^f^ Data of 36 patients was missing.

^g^ Data of 37 patients was missing.

^h^ Data of 39 patients was missing.

^I^ Data of 66 patients was missing.

^j^ GAP data of 24 patients was missing.

^k^ Data of 5 patients was missing (4 IPF, 1 other ILD).

^l^ Pack-year data of 11 ex- or current smokers was missing.

^m^ Data of two patients missing.

Abbreviations: Dg, diagnosis; DLCO, diffusion capacity for carbon monoxide; FEV1, forced expiratory volume in one second; FVC, forced vital capacity; ILD, interstitial lung disease; PFT, pulmonary function test; pred, predicted; VC, vital capacity.

The subacute progression of ILD was defined as increased fibrotic changes either in HRCT or chest x-ray compared with previous images. A subclass “respiratory symptoms without explanatory findings” was used for patients hospitalized non-electively due to acute respiratory symptoms, but whose investigations did not reveal any acute findings in clinical status, HRCT, chest x-ray or laboratory tests. A lower respiratory tract infection, e.g. pneumonia or acute bronchitis, was recorded as the reason for hospitalization if the patient had the typical symptoms of a lower respiratory tract infection (increased cough and mucus production above the normal without other reasons), with or without an elevated CRP level or a new infiltrate in chest x-ray or HRCT. The treatment periods caused by heart failure, coronary atherosclerosis, myocardial infarction or atrial fibrillation were considered to be cardiovascular (CV). Heart failure was documented as the reason for hospitalization if at least two of the following criteria were met: congestion in HRCT or chest x-ray, elevated B-type natriuretic peptide (BNP), swollen limbs or a finding compatible with heart failure in the echocardiogram. High Gender-Age-Physiology (GAP) index and GAP stage were defined as in the previous study [20]. The overall survival time was calculated from diagnosis date to death, transplantation or last follow-up date (31/8/2019). Post-hospitalization survival was determined from the first hospitalization date to death, transplantation or to last follow-up date.

### Statistical analysis

IBM SPSS Statistics for Windows, Version 25.0 (Armonk, NY: IBM Corp.) was used to perform statistical analysis and Origin(Pro), Version 2019b (OriginLab Corporation, Northampton, MA, USA) was utilized for graphs. Means and standard deviations were calculated for continuing variables, which were normally distributed. Medians and inter-quartile range (IQR) were utilized for continuing variables which were not normally distributed. Group differences of continuous variables were tested by using independent samples *t*-test and variance analyses or Mann-Whitney U-test. Differences in the categorized variables were calculated with Chi-Square or Fisher’s Exact test. Survival was estimated by using Kaplan-Meier curves and the groups were compared with each other by using log rank tests. Log rank test with a linear trend for factor levels was used, when the survival differences of patients were evaluated according to the cause of the hospitalization. Cox regression model was utilized for the assessment of risk factors for death and AE-ILD.

### Ethics

As this was a retrospective study and the majority of study subjects were deceased, no patient consent forms were gathered in accordance with Finnish legislation. The study protocol was approved by the Ethical Committee of the Northern Ostrobothnia Hospital District (statement 2/2015). Permission to use death certificates was given by Statistics Finland (Dnro: TK-53-515-15). The study was conducted in compliance with the Declaration of Helsinki.

## Results

### Patient demographics

The most common ILD type was IPF, accounting for 58% of the 237 patients. The median follow-up time of the patients was 4.7 years. The hospital mortality was 11% with no statistically significant difference between IPF and non-IPF cases. The majority of the patients (87%) had died at the end of the follow-up time, with more survivors among the non-IPF patients than in IPF. Median survival time from diagnosis in IPF patients was significantly shorter, e.g. 3.5 years, compared with non-IPF patients who had 7.8 years’ median survival (p<0.001, log rank-test). The post-hospitalization survival was also shorter in IPF patients, e.g. 1.1 years, compared with non-IPF patients with a survival time of 1.9 years (p = 0.001, log rank-test). Vital capacity (VC), but not forced vital capacity (FVC), and diffusion capacity for carbon monoxide (DLCO) in the non-IPF patients at diagnosis were higher than those of IPF patients; however, these differences could no longer be observed in the PTF results examined near the hospitalization date. Slightly more than every fourth patient (26%) had ILD diagnosed during the non-elective hospital treatment period. The median time from the ILD diagnosis date to the hospitalization date in those with a previous ILD diagnosis was more than double in non-IPF patients as compared to those with IPF, namely 5.9 years compared with 2.4 years (p<0.001). Characteristics of the study subjects are shown in Table 2 and ILD types and the number of patients having experienced an AE-ILD are presented in Table 3. Medical therapy for ILD prior to hospitalization is shown in S1 Table.

**Table 3:** Patients with interstitial lung diseases (ILD) hospitalized due to acute respiratory symptoms.

Type of ILD	N = 237	Male/Female N = 144/N = 93	AE-ILD during follow-up N = 128
	N (%)	N/N	N (% within ILD type/% within AE-ILD)
IPF	138 (58)	91 /47	79 (57/62)
Asbestosis	22 (9.2)	22/0	10 (45/8.6)
Asbestosis and RA	2 (0.8)	2/0	1 (50/0.8)
Idiopathic NSIP	19 (8.0)	5/14	10 (53/7.8)
CHP	8 (3.4)	4/4	4 (50/3.1)
CTD-ILD	33 (14)	13/20	20 (61/16)
RA	26 (11)	12/14	17 (65/13)
SSc	1 (0.4)	1/0	1 (100/0.8)
PM	1 (0.4)	0/1	0
MCTD	1 (0.4)	0/1	0
pSS	2 (0.8)	0/2	2 (100/1.6)
pSS + SLE	1 (0.4)	0/1	0
SLE	1 (0.4)	0/1	0
DIP	1 0.4)	0/1	0
Unclassifiable ILD	14 (5.9)	7/7	4 (29/3.1)

Abbreviations: AE-ILD, acute exacerbation of interstitial lung disease; CHP, chronic hypersensitivity pneumonitis; CTD-ILD, connective tissue disease-associated interstitial lung disease; DIP, desquamative interstitial pneumonia; ILD, interstitial lung disease; IPF, idiopathic pulmonary fibrosis; MCTD, mixed connective tissue disease; NSIP, non-specific interstitial pneumonia; PM, polymyositis; pSS, primary Sjögren’s syndrome; RA-ILD, rheumatoid arthritis-associated ILD; SLE, systemic lupus erythematosus; SSc, systemic scleroderma.

### Most hospitalizations were due to reasons other than AE-ILD

**Fig 2:**
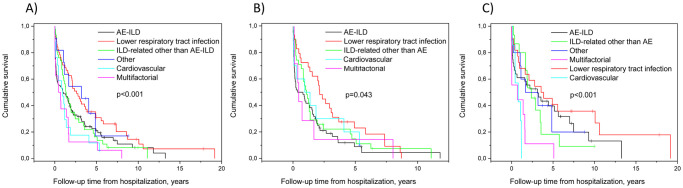
Post-hospitalization survival time was associated with the cause for hospital treatment. (A) All patients, (B) patients with idiopathic pulmonary fibrosis (IPF), (C) patients with non-IPF ILD. An ILD-related hospitalization signified a diagnosis of subacute ILD, subacute ILD progression or acute respiratory symptoms without other new, explanatory findings. Multifactorial hospitalization indicated lower respiratory tract infection concurrently with some other cause(s). Abbreviations: AE-ILD, acute exacerbation of interstitial lung disease; ILD, interstitial lung disease, IPF, idiopathic pulmonary fibrosis.

AE-ILD accounted for 96 (41%) of all hospitalizations with no statistically significant difference between IPF and non-IPF cases (Table 4). The AE-ILD was fatal in 20 patients (IPF n = 15, other ILDs n = 5). A lower respiratory tract infection (22%) and CV causes (7.2%) were the most common single causes of non-ILD-related hospitalizations. A subacute ILD progression was a more common reason for acute hospitalization among IPF patients than in their non-IPF counterparts. Non-ILD-related hospitalizations for various reasons (subcategory “other” in Table 4) were more common in non-IPF patients than in IPF. The cause for hospitalization was associated with post-hospitalization survival (Fig 2). Patients with a lower respiratory tract infection had a more favorable prognosis compared with patients with an AE-ILD (Table 5). In the subgroup of non-IPF patients with a CV or a multifactorial cause for hospitalization, the post-hospitalization survival was shorter than in patients experiencing an AE-ILD (Table 5).

**Table 4:** Causes for the first non-elective hospitalizations due to acute respiratory worsening.

Parameter, No. (%)	Total N = 237	IPF N = 138	Other ILD N = 99	P-value
AE-ILD	96 (41)	60 (44)	36 (36)	0.271
Triggered AE-ILD	8 (3.4)	3 (2.2)	5 (5.1)	0.284
No trigger	88 (37)	57 (41)	31 (31)	0.116
ILD-related hospitalization other than AE-ILD ^a^	46 (19)	31 (23)	15 (15)	0.160
Subacute ILD progression	28 (12)	23 (17)	5 (5.1)	0.006
Respiratory symptoms without explanatory findings	9 (3.8)	3 (2.2)	6 (6.1)	0.170
Diagnosis of ILD at subacute phase	9 (3.8)	5 (3.6)	4 (4.0)	1.000
Lower respiratory tract infection	51 (22)	29 (21)	22 (22)	0.823
Multifactorial ^b^	16 (6.8)	7 (5.1)	9 (9.1)	0.224
Cardiological cause	17 (7.2)	10 (7.2)	7 (7.1)	0.959
Other cause ^c^	11 (4.6)	1 (0.7)	10 (10)	0.001

^a^ Hospitalizations related to diagnosis of subacute ILD, subacute ILD progression or acute respiratory symptoms without other new, explanatory findings.

^b^ Lower respiratory tract infection concurrently with some other cause(s): cardiovascular (5 IPF, 7 other ILD), acute exacerbation of asthma (2 non-IPF ILD), acute exacerbation of COPD (1 IPF, 1 other ILD), lung cancer (1 IPF, 1 other ILD).

^c^ One non-IPF patient had pleural effusion, one non-IPF patient had acute exacerbation of chronic obstructive lung disease (COPD), one IPF patient had haemoptysis, one non-IPF patient had escherichia coli septicemia, three non-IPF patients had pulmonary embolism, one non-IPF patient had pneumothorax and aspergilloma, one non-IPF patient had bilateral pneumothorax and one non-IPF patient was suspected of experiencing an allergic reaction to the local anesthetic used during bronchoscopy procedure.

Abbreviations: AE-ILD, acute exacerbation of interstitial lung disease; ILD, interstitial lung disease, IPF, idiopathic pulmonary fibrosis.

**Table 5:** Post-hospitalization survival of the patients with AE-ILD compared with patients hospitalized due to other cause.

Parameter	Total N = 237		IPF N = 138		Other ILD N = 99	
	HR (95% CI)	P-value	HR (95%CI)	P-value	HR (95%CI)	P-value
AE-ILD	Reference		Reference		Reference	
ILD-related hospitalization other than AE-ILD ^a^	0.95 (0.65–1.39)	0.791	0.80 (0.51–1.26)	0.331	1.13 (0.57–2.25)	0.729
Lower respiratory tract infection	0.64 (0.44–0.92)	0.017	0.58 (0.36–0.92)	0.021	0.70 (0.37–1.32)	0.270
Cardiovascular cause	1.38 (0.81–2.37)	0.240	0.80 (0.39–1.63)	0.545	3.29 (1.36–7.94)	0.008
Other cause ^b^	0.64 (0.31–1.33)	0.231	0.49 (0.07–3.53)	0.475	1.04 (0.45–2.39)	0.936
Multifactorial ^c^	1.62 (0.95–2.78)	0.078	0.98 (0.44–2.18)	0.962	3.08 (1.42–6.67)	0.004

^a^ Hospitalizations related to diagnosis of subacute ILD, subacute ILD progression or acute respiratory symptoms without other new, explanatory findings.

^b^ One non-IPF patient had pleural effusion, one non-IPF patient had mild pericarditis, one IPF patient atrial fibrillation, one IPF patient had haemoptysis, one non-IPF patient had escherichia coli septicemia, two non-IPF patients had pulmonary embolism, one non-IPF patient had pneumothorax and aspergilloma, one non-IPF patient had bilateral pneumothorax and one non-IPF patient was suspected of experiencing an allergic reaction to an local anesthetic used during bronchoscopy procedure.

^c^ Two or more of the following causes occurring simultaneously: Lower respiratory tract infection, cardiovascular cause or other cause.

Abbreviations: AE-ILD, acute exacerbation of interstitial lung disease; GAP, Gender-Age-Physiology index; HR, hazard ratio.

### High GAP index was a risk factor for mortality and earlier AE-ILD

**Fig 3:**
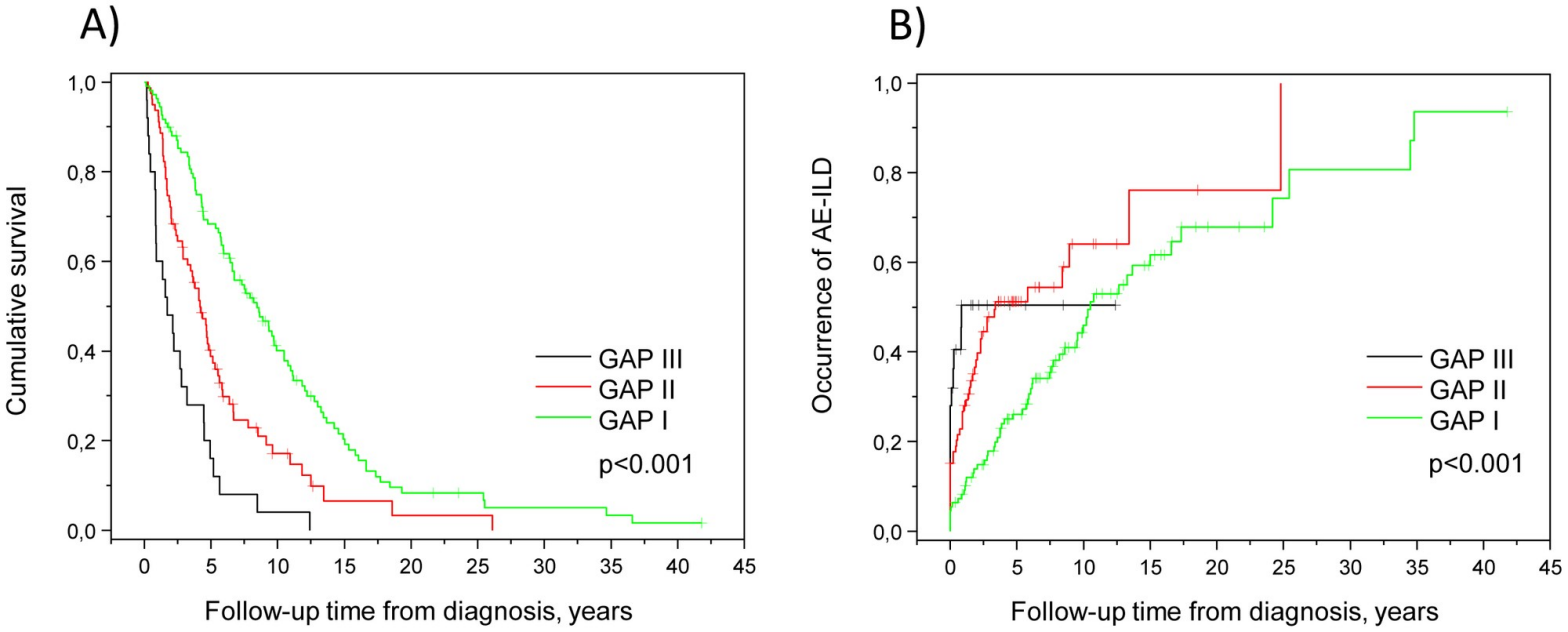
Gender-Age-Physiology index (GAP) was associated with survival time (A) and time to the first acute exacerbation of interstitial lung disease (AE-ILD) (B).

**Fig 4:**
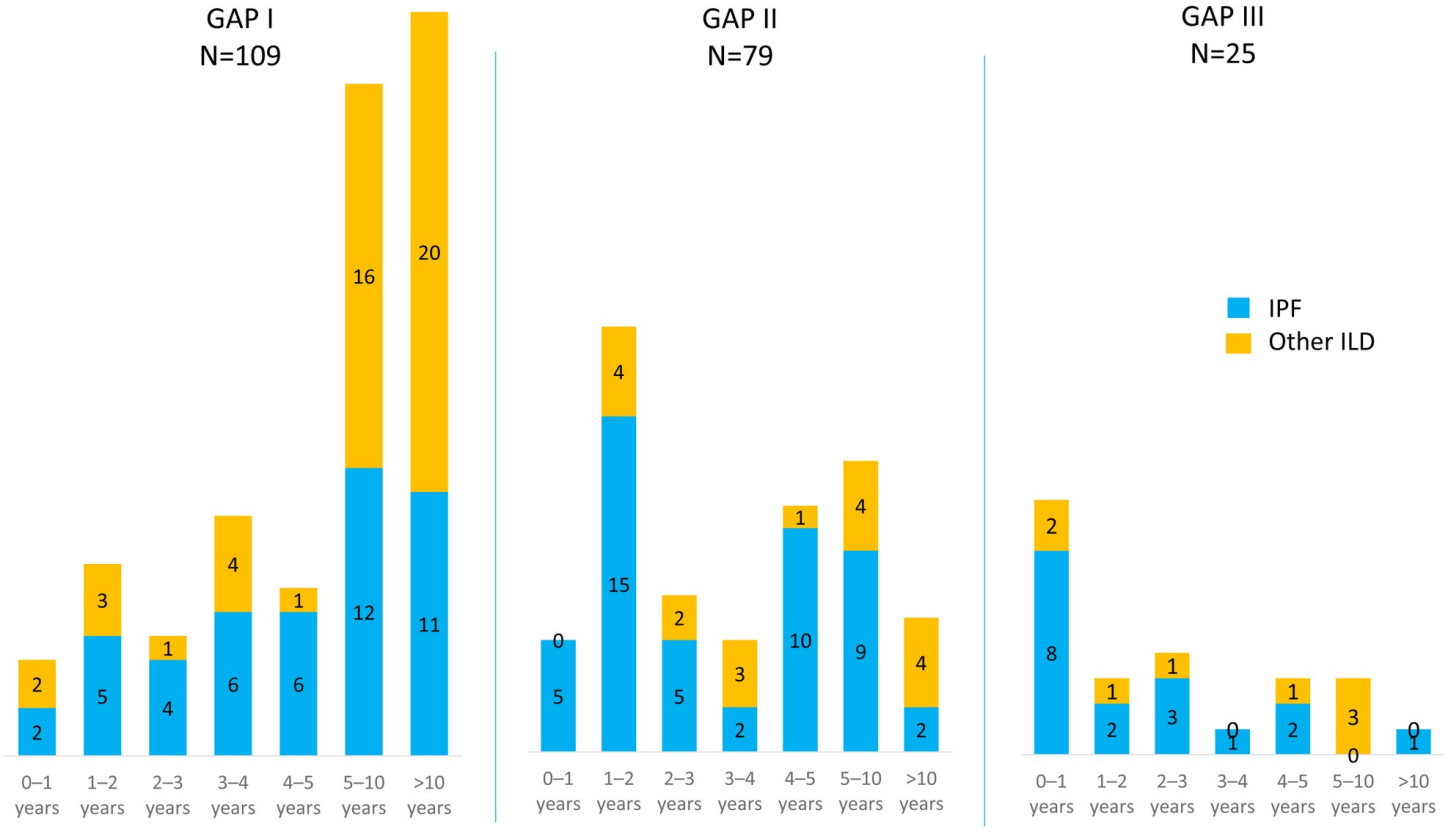
Deaths at each time point after the diagnosis according to the Gender-Age-Physiology (GAP) stages.

**Fig 5:**
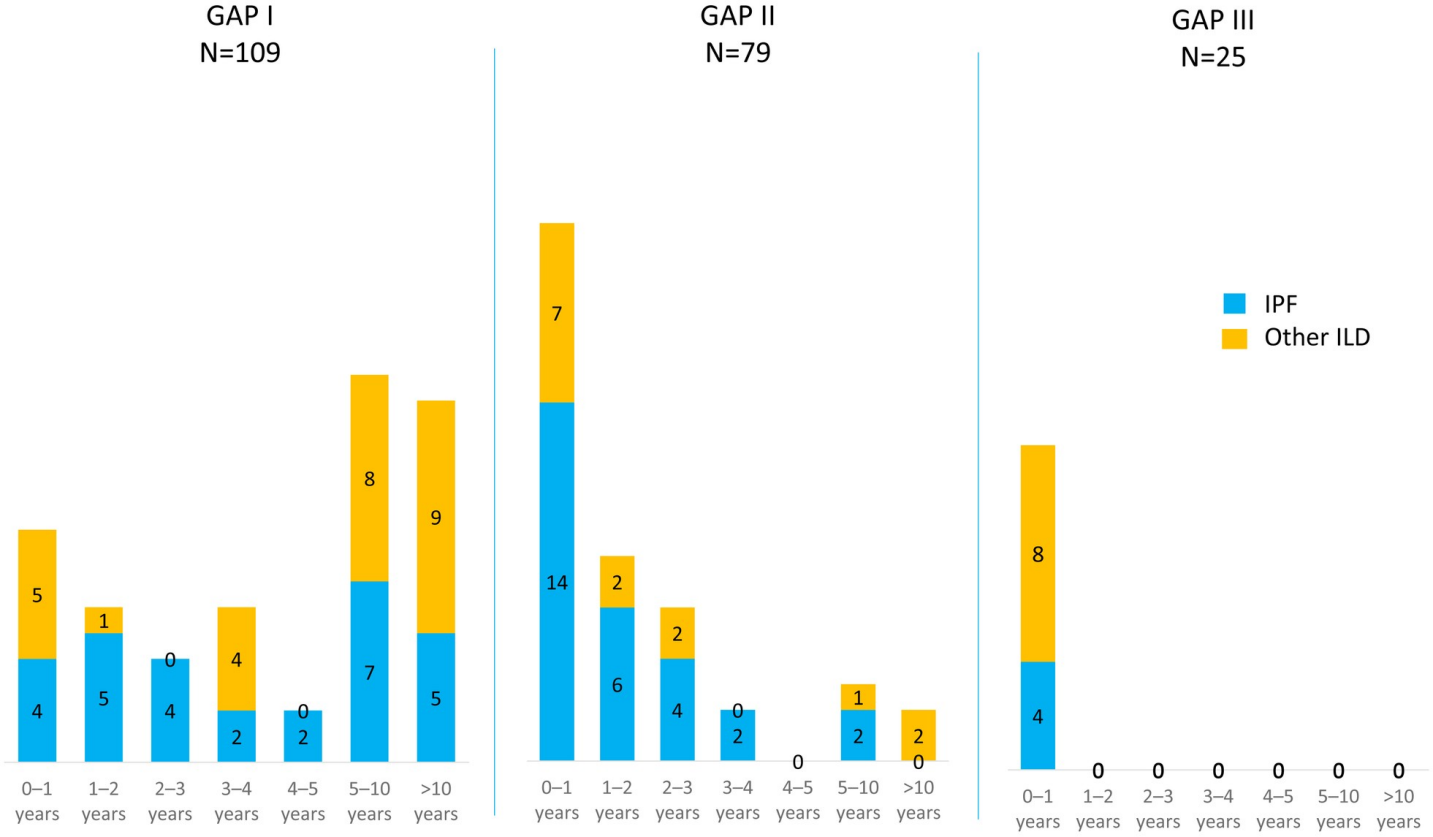
Acute exacerbations of interstitial lung disease (AE-ILD) at each time point after the diagnosis according to the Gender-Age-Physiology (GAP) stages. Only the patient’s first episode of AE-ILD is presented in charts.

A high GAP index was associated with a shorter overall survival and an earlier occurrence of AE-ILD in all patients (Fig 3, Table 6). The occurrence of deaths and AE-ILDs at different time points from diagnosis according to the GAP stages is presented in Figs 4 and 5 and in S2 Table. Causes of death are shown in S3 Table. Slightly more than half (52.6%) of the 19 patients who survived less than one year had GAP stage III. GAP stage III patients who experienced an AE-ILD had suffered this episode in less than a year after the diagnosis of ILD; in fact, none of the GAP stage III patients had their first AE-ILD later than 1 year after the diagnosis. Survival differences in the various GAP stages were also evaluated with a multivariate model involving the occurrence of AE-ILD, which had no significant impact on the result, since HR between GAP stage II and GAP stage I was 1.97 (95% CI 1.42 to 2.73, p <0.001), while HR between GAP stage III and I was 4.52 (95% CI 2.84 to 7.19, p < 0.001).

**Table 6:** The risk for death and acute exacerbations (Cox Univariate model).

Parameter	N = 237		IPF N = 138		Other ILD N = 99	
	GAP N = 213		GAP N = 120		GAP N = 93	
	HR (95% CI)	P-value	HR (95%CI)	P-value	HR (95%CI)	P-value
Risk for mortality						
GAP at diagnosis						
I	Reference		Reference		Reference	
II	1.97 (1.42–2.73)	<0.001	2.10 (1.36–3.24)	0.001	1.51 (0.87–2.63)	0.146
III	4.45 (2.80–7.07)	<0.001	3.78 (2.11–6.74)	<0.001	5.29 (2.36–11.86)	<0.001
Risk for AE-ILD						
GAP at diagnosis						
I	Reference		Reference		Reference	
II	1.96 (1.29–2.97)	0.002	1.77 (1.02–3.08)	0.041	2.05 (1.05–4.00)	0.036
III	2.81 (1.47–5.38)	0.002	2.40 (1.07–5.40)	0.034	3.03 (1.01–9.08)	0.048

Abbreviations: AE-ILD, acute exacerbation of interstitial lung disease; CI, confidence interval; GAP, Gender-Age-Physiology index; HR, hazard ratio; ILD, interstitial lung disease; IPF, idiopathic pulmonary fibrosis.

## Discussion

We have investigated data in 237 ILD patients that were hospitalized non-electively due to acute worsening of respiratory symptoms. All of the data of each patient was re-assessed in a very detailed manner. Acute exacerbations of ILD explained about 40% of the hospitalizations whereas the majority of the hospitalizations were explained by other causes. The cause for hospitalization was associated with survival time in all patients, patients with lower respiratory infection having the most favorable prognosis. A high GAP index was associated with both a shortened survival and an earlier occurrence of AE-ILDs and was a more specific indicator for earlier AE-ILDs than for deaths.

Non-IPF patients were found to have a lower GAP stage and higher VC and DLCO levels at the time of diagnosis as compared with IPF patients, indicative of a less progressed disease of non-IPF patients at baseline. However, at the time of hospitalization, the differences in VC and DLCO between IPF and non-IPF patients had disappeared. As can be seen in S4 Table, in comparison with most other studies, here the median time from diagnosis to hospitalization seemed to be longer [5, 6, 9] and patients were older [5–7, 9], even though the baseline pulmonary function test (PFT) values in our study and those of others were approximately at the same level. A Japanese study has reported higher PFT results in their investigated subjects near the first hospitalization date in comparison with our material, even though the patients in that study and our own were approximately the same age [11]. These differences may be attributable to the different genetic and ethnic backgrounds of the study subjects and also to the heterogeneity of the non-IPF patients in the above publications [5, 7, 11]. There are also some previous studies suggesting that ethnic background might influence the clinical features and disease course of the ILD patients [21, 22].

AE-ILD has been reported to be the most common individual cause for a respiratory deterioration in different types of ILDs accounting for 29–55% [5, 6, 10, 11] of respiratory hospitalizations, values consistent with our own findings. Lower respiratory tract infections were observed in 20–33% of respiratory hospitalizations in previous studies, which is in line with our result of 22% [5, 6, 10, 11]. Furthermore, a subacute progression was the reason for hospitalization in 12% of patients in our study, again in accordance with earlier investigations reporting proportions of 15% and 17% [5, 10]. Given the challenges concerning the diagnostic practices of ILD patients with acute respiratory worsening, which make retrospective studies extremely demanding to perform, the results of the previous and the present study are amazingly consistent. To be able to identify the reasons for hospitalizations retrospectively, the clinical evaluation of the patients during the hospital treatment had to be thorough e.g. involving HRCT imaging in most cases. The consistent findings of the studies might also be related to the fact that researchers in European, American and Asian hospitals have utilized the current criteria for AE-ILD, allowing them to differentiate between AE-ILDs and other causes of hospitalizations based on the same principles. So far, our study has been the only one conducted in Europe, since other studies have investigated patients originating from either the USA [5, 7, 9] or Asia [6, 11]. Thus, it does seem that the ethnic background did not exert a major impact on the reasons for respiratory worsening of ILD patients.

AE-ILD has been reported to be associated with a poor outcome in various types of ILDs [5, 6, 10, 23–26]. The treatment of AE-ILDs usually includes high doses of corticosteroids and/or other immunosuppressive therapies, even though the evidence of their efficacy is limited [12, 13]. Our results together with earlier investigations suggest that it is crucial to be meticulous in the differential diagnostics in ILD patients with acute respiratory symptoms. Based on the present results and those of other investigators, most hospitalizations were due to some reasons other than AE-ILD. Patients deserve to be treated according to the actual cause, thus avoiding the potentially harmful effects of the treatments targeted at AE-ILD.

In our study population, those patients hospitalized due to AE-ILD had a shorter survival compared with patients with a lower respiratory tract infection, the difference was more distinct in IPF patients in comparison to those with other ILDs. A similar result has also been presented by Teramachi et al. in a recent study of IPF patients, in which a respiratory tract infection was associated with a reduced 90-day post-hospitalization mortality as compared with other causes for hospitalization [10]. It can be speculated that the occurrence of a respiratory tract infection, if not fulfilling the criteria for a triggered AE-ILD, may be a marker of an ILD phenotype associated with a slow disease progression.

Interestingly, in the subgroup of non-IPF patients with a CV cause for hospitalization or a lower respiratory tract infection concurrent with some other cause, the post-hospitalization survival time was shorter than in non-IPF patients with AE-ILD. Moua et al. have observed that in non-IPF patients, an AE-ILD did not cause increased mortality in comparison with other types of hospitalizations in a multivariate model [5]. These findings may reflect the fact that AE-ILDs are less fatal for non-IPF patients than for those patients with IPF, which was also supported by our previous study, where IPF patients had a significantly shorter survival after an AE in comparison with non-IPF patients [14].

In our study, the GAP index was a risk factor for both death and AE-ILD. At least one of these findings has been described in earlier investigations [18, 27–32], even though this has not been verified in all reports [9, 33]. We also demonstrated that AE-ILDs can occur at any phase of the disease in a cohort involving both IPF and non-IPF patients, a finding which is a known feature of IPF, making the disease course difficult to predict [12, 34].

The occurrence of AE-ILD was relatively high, e.g. 54%, in our study population. In the previous studies presenting data on GAP and its effect on mortality, the prevalence of AE-ILD has varied between 20–40% [18, 22, 23], these discrepancies may be caused by different types of study protocols, e.g. we included only non-electively hospitalized patients. Our results indicate that the GAP index is a functional prognostic marker even in a cohort enriched with AE-ILDs. Nonetheless, almost half of the patients survived less than one year from diagnosis had GAP stage I or II at baseline, suggesting that GAP alone did not provide an exact prognosis for these patients, a result consistent with earlier studies presenting data on the accuracy of GAP in the prognostic evaluation of IPF patients [29, 31]. However, GAP stage III seemed to be quite a specific predictor of early AE-ILDs, since all the patients with GAP stage III disease at diagnosis experienced an AE-ILD within 1 year after the diagnosis, whereas those GAP stage III patients who survived the first year without an AE-ILD did not experience the episode later. To our knowledge, there is no earlier published data demonstrating similar findings.

In Finland pirfenidone received a recommendation for reimbursement in 2013 and nintedanib in 2015 for the treatment of the patients with IPF by the Social Insurance Institution of Finland (Kela). According to the open database of Kela, about 90 IPF patients had received a reimbursement for antifibrotic drugs in OUH and OH by the end of 2017 when only 13 patients in our study had been taking pirfenidone or nintedanib prior to hospitalization (S1 Table) [35]. Since only the patients with acute hospitalizations, but not all, were included in our study, the results of Kela database are more trustworthy about the use of antifibrotic drugs in our hospital area than the numbers of the present study. Thus, one can speculate that perhaps the patients who were not being treated with antifibrotic therapy were more often hospitalized than those receiving this form of drug treatment.

Our study has some limitations that are common in retrospective studies, namely some missing patient data and the possible incorrect use of ICD-10 diagnosis codes by clinicians, which might have led to the exclusion of a few potential cases. Furthermore, a radiologist did not re-evaluate the HRCT scans of the patients especially for this research, which can also be regarded as a limitation of this study. Nevertheless, the re-evaluation of the HRCTs was performed for the majority of the patients in MDD by thoracic radiologists as a part of the routine clinical practice. The small numbers of patients with a certain non-IPF ILD subtype can also be considered a limitation of our study. However, combining different progressive ILD subtypes together to a group called “progressive fibrosing interstitial lung diseases” (PF-ILD) has recently become more common [36–40] and we believe that our study may provide relevant novel important information on the disease course of PF-ILDs.

The careful and systematic evaluation of the data as well as the re-classification of ILD types according to the current international guidelines are the strengths of our study. It should be noted that differential diagnostics in hospitalized patients with progressed ILD would be challenging even in a prospective study protocol, because AE-ILD, respiratory infections and heart failure often share similar and even concurrent clinical and radiological features.

## Conclusions

To conclude, although AE-ILD was the most common single cause for hospitalization, a relatively large proportion of hospitalizations, e.g. 59%, was attributable to causes other than AE-ILD. In view of the potentially harmful effects of the therapies targeted at managing an AE-ILD, clinicians should be aware that there are multiple possible etiologies in addition to an AE-ILD when treating ILD patients with acute respiratory worsening; some of them may be potentially even more serious for patients than an AE.

## Supporting information

S1 Table Pharmacological therapy for idiopathic pulmonary fibrosis (IPF) and other interstitial lung disease (ILD) prior to the hospital treatment. Data are presented as numbers of patients (%) or median (interquartile range). ^a^ The total number of patients treated with corticosteroid monotherapy or combination therapy either because of ILD or other disease. Cs, corticosteroid; ILD, interstitial lung disease; IPF, idiopathic pulmonary fibrosis; NAC, N-acetylcysteine.
(PDF)

S2 Table Deaths and patients with an acute exacerbation of ILD in different Gender-Age-Physiology (GAP) stages. Data are presented as the number of patients (% of all patients within the GAP stage / % of cases (either deaths or AE-ILDs) within the follow-up time category). Only the patients for whom GAP data was available were included in the analysis; GAP data of 24 patients was missing. There were 16 patients in GAP I, 13 patients in GAP II and 0 patients in GAP III alive at the end of the follow-up time. Of these still living patients, 8 had a follow-up time less than 10 years. ^a^ Of the patients having experienced multiple AE-ILDs, only the first AE-ILD is recorded. Abbreviations: AE-ILD, acute exacerbation of interstitial lung disease. AE-ILD, acute exacerbation of interstitial lung disease; GAP, gender-age-physiology index, ILD, interstitial lung disease.
(PDF)

S3 Table Underlying and immediate causes for death in idiopathic pulmonary fibrosis (IPF) and other interstitial lung disease (ILD) patients. Seven patients with lung transplantation were excluded from the analysis. ^a^ 2 patients had Morbus Alzheimer, 1 diabetes mellitus, 1 Morbus Parkinson, 1 chronic obstructive lung disease and 1 patient was drowned. ^b^ 1 patient had staphylococcus aureus septicemia and 1 patient had septicemia caused by a gram-negative rod bacterium. ^c^ 1 patient had Morbus Alzheimer, 1 an epileptic seizure, 1 an acute kidney injury.
(PDF)

S4 Table Clinical features of ILD patients in the previous and the present studies at baseline and during hospitalization. Abbreviations: AE, acute exacerbation of ILD; c-IIP, chronic idiopathic interstitial pneumonia; CTD-ILD, connective tissue disease-associated interstitial lung disease; DLCO, diffusion capacity for carbon monoxide; FVC, forced vital capacity; ILD, interstitial lung disease; IQR, interquartile range; IPF, idiopathic pulmonary fibrosis; NA, not applicable; SD, standard deviation.
(PDF)

S1 Appendix Anonymized patient data. (XLSX)

## References

[1] Pedraza-Serrano F, López de Andrés A, Jiménez-García R, Jiménez-Trujillo I, Hernández-Barrera V, Sánchez-Muñoz G, et al Retrospective observational study of trends in hospital admissions for idiopathic pulmonary fibrosis in Spain (2004–2013) using administrative data. BMJ Open. 2017;7:e013156 10.1136/bmjopen-2016-013156. 28193850 PMC5318548

[2] Yu YF, Wu N, Chuang C, Wang R, Pan X, Benjamin NN, et al Patterns and Economic Burden of Hospitalizations and Exacerbations Among Patients Diagnosed with Idiopathic Pulmonary Fibrosis. J Manag Care Spec Pharm. 2016;22: 414–23 10.18553/jmcp.2016.22.4.414. 27023695 PMC10398274

[3] Cottin V, Schmidt A, Catella L, Porte F, Fernandez-Montoya C, Le Lay K, et al Burden of Idiopathic Pulmonary Fibrosis Progression: A 5-Year Longitudinal Follow-Up Study. PLoS ONE. 2017;12:e0166462 10.1371/journal.pone.0166462. 28099456 PMC5242514

[4] Wälscher J, Witt S, Schwarzkopf L, Kreuter M. Hospitalisation patterns of patients with interstitial lung disease in the light of comorbidities and medical treatment—a German claims data analysis. Respir Res. 2020;21:73 10.1186/s12931-020-01335-x. 32216792 PMC7098099

[5] Moua T, Westerly BD, Dulohery MM, Daniels CE, Ryu JH, Lim KG. Patients with fibrotic interstitial lung disease hospitalized for acute respiratory worsening: A large cohort analysis. Chest. 2016;149:1205–14. 26836940 10.1016/j.chest.2015.12.026

[6] Song JW, Hong SB, Lim CM, Koh Y, Kim DS. Acute exacerbation of idiopathic pulmonary fibrosis: incidence, risk factors and outcome. Eur Respir J. 2011;37:356–63 10.1183/09031936.00159709. 20595144

[7] Ratwani AP, Ahmad KI, Barnett SD, Nathan SD, Brown AW. Connective tissue disease-associated interstitial lung disease and outcomes after hospitalization: A cohort study. Respir Med. 2019;154:1–5 10.1016/j.rmed.2019.05.020. 31176795

[8] Behr J, Kreuter M, Hoeper MM, Wirtz H, Klotsche J, Koschel D, et al Management of patients with idiopathic pulmonary fibrosis in clinical practice: the INSIGHTS-IPF registry. Eur Respir J. 2015;46:186–96 10.1183/09031936.00217614. 25837040 PMC4486374

[9] Brown AW, Fischer CP, Shlobin OA, Buhr RG, Ahmad S, Weir NA, et al Outcomes after hospitalization in idiopathic pulmonary fibrosis: a cohort study. Chest. 2015;147:173–9 https://doi.org/S0012-3692(15)30247-6. 10.1378/chest.13-2424 25188694

[10] Teramachi R, Kondoh Y, Kataoka K, Taniguchi H, Matsuda T, Kimura T, et al Outcomes with newly proposed classification of acute respiratory deterioration in idiopathic pulmonary fibrosis. Respir Med. 2018;143:147–52 10.1016/j.rmed.2018.09.011. 30261987

[11] Yamazaki R, Nishiyama O, Saeki S, Sano H, Iwanaga T, Tohda Y. Characteristics of patients with chronic idiopathic interstitial pneumonia undergoing repeated respiratory-related hospitalizations: A retrospective cohort study. PLoS ONE. 2020;15(4):e0232212 10.1371/journal.pone.0232212. 32330195 PMC7182228

[12] Collard HR, Ryerson CJ, Corte TJ, Jenkins G, Kondoh Y, Lederer DJ, et al Acute exacerbation of idiopathic pulmonary fibrosis. An international working group report. Am J Respir Crit Care Med 2016;194:265–75 10.1164/rccm.201604-0801CI. 27299520

[13] Kolb M, Bondue B, Pesci A, Miyazaki Y, Song JW, Bhatt NY, et al Acute exacerbations of progressive-fibrosing interstitial lung diseases. Eur Respir Rev 2018;27:180071 https://doi.org.10.1183/16000617.0071-2018. 30578331 10.1183/16000617.0071-2018 PMC9488799

[14] Salonen J, Purokivi M, Bloigu R, Kaarteenaho R. Prognosis and causes of death of patients with acute exacerbation of fibrosing interstitial lung diseases. BMJ Open Resp Res. 2020;7:e000563 10.1136/bmjresp-2020-000563 32265195 PMC7254157

[15] Raghu G, Remy-Jardin M, Myers JL, Richeldi L, Ryerson CJ, Lederer DJ, et al Diagnosis of Idiopathic Pulmonary Fibrosis. An Official ATS/ERS/JRS/ALAT Clinical Practice Guideline. Am J Respir Crit Care Med. 2018;198:e44–68 10.1164/rccm.201807-1255ST. 30168753

[16] Travis WD, Costabel U, Hansell DM, King TE, Lynch DA, Nicholson AG, et al An official American Thoracic Society/European Respiratory Society statement: Update of the international multidisciplinary classification of the idiopathic interstitial pneumonias. Am J Respir Crit Care Med 2013;188:733–48 10.1164/rccm.201308-1483ST. 24032382 PMC5803655

[17] Miller MR, Hankinson J, Brusasco V, Burgos F, Casaburi R, Coates A, et al ATS/ERS task force: stantardisation of lung function testing. Standardisation of spirometry. Eur Respir J 2005;26:319–38. 10.1183/09031936.05.00034805 16055882

[18] Miller MR, Crapo R, Hankinson J, Brusasco V, Burgos F, Casaburi R, et al ATS/ERS task force: Standardisation of lung function testing. General considerations for lung function testing. Eur Respir J 2005;26:153–61. 10.1183/09031936.05.00034505 15994402

[19] Viljanen AA, Halttunen PK, Kreus K-E, Viljanen BC. Spirometric studies in non-smoking, healthy adults. Scand J Clin Lab Invest. 1982;159:5–20. 6957974

[20] Ley B, Ryerson CJ, Vittinghoff E, Ryu JH, Tomassetti S, Lee JS, et al A multidimensional index and staging system for idiopathic pulmonary fibrosis. Ann Intern Med. 2012;156:684–91 10.7326/0003-4819-156-10-201205150-00004. 22586007

[21] Swigris JJ, Olson AL, Huie TJ, Fernandez-Perez ER, Solomon J, Sprunge D, et al Ethnic and racial differences in the presence of idiopathic pulmonary fibrosis at death. Respir Med. 2012;106:588–93. 10.1016/j.rmed.2012.01.002. 22296740 PMC3294009

[22] Chan C, Ryerson CJ, Dunne JV, Wilcox PG. Demographic and clinical predictors of progression and mortality in connective tissue disease-associated interstitial lung disease: a retrospective cohort study. BMC Pulm Med. 2019;19:192 10.1186/s12890-019-0943-2 31672127 PMC6824100

[23] Kakugawa T, Sakamoto N, Sato S, Yura H, Harada T, Nakashima S, et al Risk factors for an acute exacerbation of idiopathic pulmonary fibrosis. Respir Res. 2016;17:79 10.1186/s12931-016-0400-1. 27401332 PMC4940941

[24] Yamakawa H, Sato S, Tsumiyama E, Nishizawa T, Kawabe R, Oba T, et al Predictive factors of mortality in rheumatoid arthritis-associated interstitial lung disease analysed by modified HRCT classification of idiopathic pulmonary fibrosis according to the 2018 ATS/ERS/JRS/ALAT criteria. J Thorac Dis. 2019;11:5247–57 10.21037/jtd.2019.11.73. 32030242 PMC6987998

[25] Liang J, Cao H, Ke Y, Sun C, Chen W, Lin J. Acute exacerbation of interstitial lung disease in adult patients with idiopathic inflammatory myopathies: A retrospective case-control study. Front Med. 2020;7:12 10.3389/fmed.2020.00012. 32083087 PMC7005087

[26] Okamoto M, Fujimoto K, Sadohara J, Furuya K, Kaieda S, Miyamura T, et al A retrospective cohort study of outcome in systemic sclerosis-associated interstitial lung disease. Respir Investig. 2016;54:445–53 10.1016/j.resinv.2016.05.004. 27886856

[27] Kawamura K, Ichikado K, Ichiyasu H, Anan K, Yasuda Y, Suga M, et al Acute exacerbation of chronic fibrosing interstitial pneumonia in patients receiving antifibrotic agents: incidence and risk factors from real-world experience. BMC Pulm Med. 2019;19:113 10.1186/s12890-019-0880-0. 31238929 PMC6593518

[28] Suzuki A, Kondoh Y, Brown KK, Johkoh T, Kataoka K, Fukuoka J, et al Acute exacerbations of fibrotic interstitial lung diseases. Respirology. 2020;25:525–34 10.1111/resp.13682. 31426125

[29] Lee SH, Park JS, Kim SY, Kim DS, Kim YW, Chung MP, et al Comparison of CPI and GAP models in patients with idiopathic pulmonary fibrosis: a nationwide cohort study. Sci Rep. 2018;8:4784 10.1038/s41598-018-23073-3. 29555917 PMC5859191

[30] Nurmi HM, Purokivi MK, Karkkainen MS, Kettunen HP, Selander TA, Kaarteenaho RL. Are risk predicting models useful for estimating survival of patients with rheumatoid arthritis-associated interstitial lung disease?. BMC Pulm Med. 2017;17:16–2 10.1186/s12890-016-0358-2. 28086844 PMC5237199

[31] Kärkkäinen M, Kettunen H, Nurmi H, Selander T, Purokivi M, Kaarteenaho R. Comparison of disease progression subgroups in idiopathic pulmonary fibrosis. BMC Pulm Med. 2019;19:228 10.1186/s12890-019-0996-2. 31783748 PMC6883511

[32] Jo HE, Glaspole I, Moodley Y, Chapman S, Ellis S, Goh N, et al Disease progression in idiopathic pulmonary fibrosis with mild physiological impairment: analysis from the Australian IPF registry. BMC Pulm Med. 2018;18: 19 10.1186/s12890-018-0575-y. 29370786 PMC5785886

[33] Atsumi K, Saito Y, Kuse N, Kobayashi K, Tanaka T, Kashiwada T, et al Prognostic factors in the acute exacerbation of idiopathic pulmonary fibrosis: A retrospective single-center study. Intern Med. 2018;57:655–61 10.2169/internalmedicine.9331-17. 29151518 PMC5874335

[34] Kondoh Y, Cottin V, Brown KK. Recent lessons learned in the management of acute exacerbation of idiopathic pulmonary fibrosis. Eur Respir Rev. 2017;26:170050 10.1183/16000617.0050-2017 28954766 PMC9488992

[35] Kela—The Social Insurance Institution of Finland [cited 16 October 2020]. Database: Existing, new and withdrawn entitlements to reimbursement of drug expenses. http://raportit.kela.fi/ibi_apps/WFServlet.

[36] Flaherty KR, Wells AU, Cottin C, Devaraj A, Walsh SLF, Inoue Y, et al Nintedanib in progressive fibrosing interstitial lung diseases. N Engl J Med. 2019;381:1718–27. 10.1056/NEJMoa1908681 31566307

[37] Brown KK, Martinez FJ, Walsh SLF, Thannickal VJ, Prasse A, Schlenker-Herceg R, et al The natural history of progressive fibrosing interstitial lung diseases. Eur Respir J. 2020;55:2000085 10.1183/13993003.00894-2020. 32217654 PMC7315005

[38] George PM, Spagnolo P, Kreuter M, Altinisk G, Bonifazi M, Martinez FJ, et al Progressive fibrosing interstitial lung disease: clinical uncertainties, consensus recommendations, and research priorities. Lancet Respir Med. 2020;8:925–34. 10.1016/S2213-2600(20)30355-6 32890499

[39] Gibson CD, Kugler MC, Deshwal H, Munger JS, Condos R. Advances in targeted therapy for progressive fibrosing interstitial lung disease. Lung. 2020;198:597–608. 10.1007/s00408-020-00370-1. 32591895

[40] Wongkarnjana A, Scallan C, Kolb MRJ. Progressive fibrosing interstitial lung disease: treatable treaits and therapeutic strategies. Curr Opin Pulm Med. 2020;26:436–42. 10.1097/MCP.0000000000000712 32657838

