# Supplementary material for: Causes of acute respiratory hospitalizations predict survival in fibrosing interstitial lung diseases
Source: PLoS One. 2020 Nov 30;15(11):e0242860. doi: 10.1371/journal.pone.0242860 (PMC7703970; doi:10.1371/journal.pone.0242860)
Supplement: S1 Table — Data are presented as numbers of patients (%) or median (interquartile range). a The total number of patients treated with corticosteroid monotherapy or combination therapy either because of ILD or other disease. Cs, corticosteroid; ILD, interstitial lung disease; IPF, idiopathic pulmonary fibrosis; NAC, N-acetylcysteine. (PDF) [file pone.0242860.s001.pdf]

S1 Table. Pharmacological therapy for idiopathic pulmonary fibrosis (IPF) and other interstitial lung disease (ILD) prior to the hospital treatment.

Pharmacological therapy	Total N=237	IPF N=138	Other ILD N=99	P-value
Monotherapy for ILD				
Corticosteroid	42 (17.7)	19 (13.8)	23 (23.2)	0.060
Pirfenidone	9 (3.8)	9 (6.5)	0	0.011
Nintedanib	2 (0.8)	2 (1.4)	0	0.511
Azathioprine	1 (0.4)	1 (0.7)	0	>0.999
Corticosteroid, NAC and azathioprine	14 (5.9)	13 (9.4)	1 (1.0)	0.007
Combined with corticosteroid				
Azathioprine	7 (3.0)	2 (1.4)	5 (5.1)	0.132
NAC	1 (0.4)	1 (0.7)	0	>0.999
Cyclophosphamide	1 (0.4)	0	1 (1.0)	0.418
Pirfenidone	1 (0.4)	1 (0.7)	0	>0.999
Nintedanib	1 (0.4)	1 (0.7)	0	>0.999
Cs for some indication other than ILD	12 (5.1)	3 (2.2)	9 (9.1)	0.017
Cs and azathioprine for some indication other than ILD	1 (0.4)	0	1 (1.0)	0.418
Corticosteroid [a]	80 (33.8)	40 (29.0)	40 (40.4)	0.067
No medical treatment for ILD	158 (66.7)	89 (64.5)	69 (69.7)	0.402

Data are presented as numbers of patients (%) or median (interquartile range). [a] The total number of patients treated with corticosteroid monotherapy or combination therapy either because of ILD or other disease. Cs, corticosteroid; ILD, interstitial lung disease; IPF, idiopathic pulmonary fibrosis; NAC, N-acetylcysteine.
