# Supplementary material for: Causes of acute respiratory hospitalizations predict survival in fibrosing interstitial lung diseases
Source: PLoS One. 2020 Nov 30;15(11):e0242860. doi: 10.1371/journal.pone.0242860 (PMC7703970; doi:10.1371/journal.pone.0242860)
Supplement: S2 Table — Data are presented as the number of patients (% of all patients within the GAP stage / % of cases (either deaths or AE-ILDs) within the follow-up time category). Only the patients for whom GAP data was available were included in the analysis; GAP data of 24 patients was missing. There were 16 patients in GAP I, 13 patients in GAP II and 0 patients in GAP III alive at the end of the follow-up time. Of these still living patients, 8 had a follow-up time less than 10 years. a Of the patients having experienced multiple AE-ILDs, only the first AE-ILD is recorded. Abbreviations: AE-ILD, acute exacerbation of interstitial lung disease. AE-ILD, acute exacerbation of interstitial lung disease; GAP, gender-age-physiology index, ILD, interstitial lung disease. (PDF) [file pone.0242860.s002.pdf]

S2 Table. Deaths and patients with an acute exacerbation of ILD in different Gender-Age-Physiology (GAP) Stages.

GAP at diagnosis N=213	Numbers of deaths at each time point from the diagnosis date							
	Years 0-1	Years 1-2	Years 2-3	Years 3-4	Years 4-5	Years 5-10	Years >10	All deaths
I N=109	4 (3.7/21.1)	8 (7.3/26.7)	5 (4.6/31.3)	10 (9.2/62.5)	7 (6.4/33.3)	28 (25.7/63.6)	31 (28.4/81.6)	93 (85.3/50.5)
II N=79	5 (6.3/26.3)	19 (24.0/63.3)	7 (8.9/43.8)	5 (6.3/31.3)	11 (13.9/43.8)	13 (16.4/29.5)	6 (7.6/15.8)	66 (83.5/35.9)
III N=25	10 (40.0/52.6)	3 (12.0/10.0)	4 (16.0/25.0)	1 (4.0/6.3)	3 (12.0/14.3)	3 (12.0/6.8)	1 (4.0/2.6)	25 (100/13.6)
Total Deaths (% all deaths)	19 (10.3)	30 (16.3)	16 (8.7)	16 (8.7)	21 (11.4)	44 (23.9)	38 (20.7)	184
GAP at diagnosis N=213	Numbers of patients having experienced an AE-ILD at each time point from the diagnosis date							
	Years 0-1	Years 1-2	Years 2-3	Years 3-4	Years 4-5	Years 5-10	Years >10	All AE-ILDs
I N=109	9 (8.3/21.4)	6 (5.5/42.9)	4 (3.7/40.0)	6 (13.1/75.0)	2 (1.8/100)	15 (13.8/83.3)	14 (12.8/87.5)	56 (51.4/50.9)
II N=79	21 (26.6/50.0)	8 (15.6/57.1)	6 (7.6/60.0)	2 (11.4/25.0)	0	3 (3.8/16.7)	2 (2.5/12.5)	42 (53.2/38.2)
III N=25	12 (48.0/28.6)	0	0	0	0	0	0	12 (48.0/10.9)
Total AE-ILDs [a] (% all AEs)	42 (38.2)	14 (12.7)	10 (9.1)	8 (7.3)	2 (1.8)	18 (16.4)	16 (14.5)	110

Data are presented as the number of patients (% of all patients within the GAP stage / % of cases (either deaths or AE-ILDs) within the follow-up time category). Only the patients with GAP data available were included in this analysis. GAP data of 24 patients was missing. There were 16 patients in GAP I, 13 patients in GAP II and 0 patients in GAP III alive at the end of the follow-up time. Of these patients alive, 8 had a follow-up time less than 10 years. [a] Of the patients having experienced multiple AE-ILDs, only the first AE-ILD is recorded. Abbreviations: AE-ILD, acute exacerbation of interstitial lung disease. AE-ILD, acute exacerbation of interstitial lung disease; GAP, gender-age-physiology index, ILD, interstitial lung disease.
