# Supplementary material for: Causes of acute respiratory hospitalizations predict survival in fibrosing interstitial lung diseases
Source: PLoS One. 2020 Nov 30;15(11):e0242860. doi: 10.1371/journal.pone.0242860 (PMC7703970; doi:10.1371/journal.pone.0242860)
Supplement: S3 Table — Seven patients with lung transplantation were excluded from the analysis. a 2 patients had Morbus Alzheimer, 1 diabetes mellitus, 1 Morbus Parkinson, 1 chronic obstructive lung disease and 1 patient was drowned. b 1 patient had staphylococcus aureus septicemia and 1 patient had septicemia caused by a gram-negative rod bacterium. c 1 patient had Morbus Alzheimer, 1 an epileptic seizure, 1 an acute kidney injury. (PDF) [file pone.0242860.s003.pdf]

S3 Table. Underlying and immediate causes of death in idiopathic pulmonary fibrosis (IPF) and other interstitial lung disease (ILD) patients.

	Total N=198	IPF N=122	Other ILD N=76	P value
Underlying cause of death				
Interstitial lung disease	142 (72)	97 (80)	45 (59)	0.002
Cardiovascular disease	37 (19)	15 (12)	22 (29)	0.003
Lung cancer	6 (3)	4 (3)	2 (3)	>0.999
Pulmonary embolism	2 (1)	0	2 (3)	0.146
Other cancer	5 (3)	2 (2)	3 (4)	0.374
Other reason [a]	6 (3)	4 (3)	2 (3)	>0.999
Immediate cause of death				
Interstitial lung disease	79 (40)	58 (48)	21 (28)	0.005
Lower respiratory tract infection	52 (26)	32 (26)	20 (26)	>0.999
Acute exacerbation of ILD or ARDS	12 (6)	7 (6)	5 (7)	>0.999
Ischemic heart disease	30 (15)	15 (12)	15 (20)	0.156
Heart failure	8 (4)	2 (2)	6 (8)	0.056
Lung cancer	5 (3)	3 (3)	2 (3)	>0.999
Other cancer	3 (2)	1 (1)	2 (3)	0.560
Other infection [b]	2 (1)	1 (1)	1 (1)	>0.999
Other [c]	3 (2)	2 (2)	1 (1)	>0.999
Pulmonary embolism	4 (2)	1 (1)	3 (4)	0.159

Seven patients with lung transplantation were excluded from the analysis. [a] 2 patients had Morbus Alzheimer, 1 diabetes mellitus, 1 Morbus Parkinson, 1 chronic obstructive lung disease and 1 patient was drowned. [b] 1 patient had staphylococcus aureus septicemia and 1 patient had septicemia caused by a gram-negative rod bacterium. [c] 1 patient had Morbus Alzheimer, 1 an epileptic seizure, 1 an acute kidney injury.
