# Supplementary material for: Causes of acute respiratory hospitalizations predict survival in fibrosing interstitial lung diseases
Source: PLoS One. 2020 Nov 30;15(11):e0242860. doi: 10.1371/journal.pone.0242860 (PMC7703970; doi:10.1371/journal.pone.0242860)
Supplement: S4 Table — Abbreviations: AE, acute exacerbation of ILD; c-IIP, chronic idiopathic interstitial pneumonia; CTD-ILD, connective tissue disease-associated interstitial lung disease; DLCO, diffusion capacity for carbon monoxide; FVC, forced vital capacity; ILD, interstitial lung disease; IQR, interquartile range; IPF, idiopathic pulmonary fibrosis; NA, not applicable; SD, standard deviation. (PDF) [file pone.0242860.s004.pdf]

S4 Table. Clinical features of ILD patients in the previous and the present studies at baseline and during hospitalization.

Characteristic	Current study	Moua et al, 2016, USA [5]	Song et al, 2011, South Korea[6]	Teramachi et al, 2018, Japan [10]	Ratwani et al, 2019, USA [7]	Brown et al, 2015, USA [9]	Yamazaki et al, 2020, Japan [11]
Material	IPF and other ILD	IPF and other ILD	IPF	IPF	CTD-ILD	IPF	IPF and c-IIP
Median time from ILD diagnosis to hospitalization, excluding first time diagnosis (months, range)	40.9 (0.07-417.6)	17.3	Mean±SD: AE 16.5±24.5 Infection 29.5±34.7	NA	NA	Respiratory hospitalization: Mean (range) 6.3 (0-80.1)	NA
Age at diagnosis (mean ± SD)	69±11	67.5±11	AE 64.3±8.9 Infection 63.7±8.6	Median (IQR): 68.0 (63.0-72.0)	57±13	Non-respiratory hospitalization: 64.6±9.8 Respiratory hospitalization: 62.8 ±113	NA
Age at hospitalization (mean ± SD)	73±9.7	69±11.5	AE 65.3±7.9 Infection 66.1±7.6	AE 73.0 (68.0-78.0) Other 69.0 (63.0-73.0)	NA	NA	74.8±7.3
Male (%)	61	66	AE 76.7 Infection 81.1	84	37	Non-respiratory hospitalization: 76.5 Respiratory hospitalization: 69	73
FVC at diagnosis (mean ± SD)	74±16	61.8±18.2	AE 72.0±15.7 Infection 75.5 ±18.5	Median (IQR) 76.2 (67.0-87.2)	58±23	Non-respiratory hospitalization: 63.6±18.3 Respiratory hospitalization: 56.4±18.1	NA
DLCO at diagnosis (mean ± SD)	53±18	40.8±15.4	AE 62.2±19.3 Infection 61.2±18.0	Median (IQR) 53.3 (44.1-64.7)	38±16	Non-respiratory hospitalization: 43.8±14.2 Respiratory hospitalization: 36.6±19.1	NA

FVC at hospitalization (mean ± SD)	63±18	NA	NA	NA	NA	NA	73.5±22.4
DLCO at hospitalization (mean ± SD)	41±15	NA	NA	NA	NA	NA	65.0±20.8

Abbreviations: AE, acute exacerbation of ILD; c-IIP, chronic idiopathic interstitial pneumonia; CTD-ILD, connective tissue disease-associated interstitial lung disease; DLCO, diffusion capacity for carbon monoxide; FVC, forced vital capacity; ILD, interstitial lung disease; IQR, interquartile range; IPF, idiopathic pulmonary fibrosis; NA, not applicable; SD, standard deviation.
